# Stimulus-Specific Adaptation Decreases the Coupling of Spikes to LFP Phase

**DOI:** 10.3389/fncir.2019.00044

**Published:** 2019-07-03

**Authors:** Mohsen Parto Dezfouli, Mohammad Zarei, Mehran Jahed, Mohammad Reza Daliri

**Affiliations:** ^1^Neuroscience and Neuroengineering Research Laboratory, Biomedical Engineering Department, School of Electrical Engineering, Iran University of Science and Technology, Tehran, Iran; ^2^Department of Electrical Engineering, Sharif University of Technology, Tehran, Iran

**Keywords:** stimulus-specific adaptation, spike-phase coupling (SPC), primary auditory cortex, local field potential (LFP), multi-unit activity (MUA)

## Abstract

Stimulus repetition suppresses the neural activity in different sensory areas of the brain. This mechanism of so-called stimulus-specific adaptation (SSA) has been observed in both spiking activity and local field potential (LFP) responses. However, much remains to be known about the effect of SSA on the spike–LFP relation. In this study, we approached this issue by investigating the spike-phase coupling (SPC) in control and adapting paradigms. For the control paradigm, pure tones were presented in a random unbiased sequence. In the adapting paradigm, the same stimuli were presented in a random pattern but it was biased to an adapter stimulus. In fact, the adapter occupied 80% of the adapting sequence. During the tasks, LFP and multi-unit activity were recorded simultaneously from the primary auditory cortex of 15 anesthetized rats. To clarify the effect of adaptation on the relation between spike and LFP responses, the SPC of the adapter stimulus in these two paradigms was evaluated. Here, we employed phase locking value method for calculating the SPC. Our data show a strong coupling of spikes to LFP phase most prominently in beta band. This coupling was observed to decrease in the adapting condition compared to the control one. Importantly, we found that adaptation reduces spikes dominantly from the preferred phase of LFP in which spikes are more likely to be present there. As a result, the preferred phase of LFP may play a key role in coordinating neuronal spiking activity in neural adaptation mechanism. This finding is important for interpretation of the underlying neural mechanism of adaptation and also can be used in the context of the network and related connectivity models.

## Introduction

Neural synchrony/desynchrony has been targeted of many recent brain studies and has deeply influenced modern knowledge in various functions, such as sensory coding, decision making, working memory, and selective attention (Eckhorn and Obermueller, 1993; Baker et al., 1999; Cutsuridis and Hasselmo, 2011; Muthukumaraswamy, 2011; Li et al., 2014; Mendoza-Halliday et al., 2014; Ruff and Cohen, 2014; Fazlali et al., 2016; Ding et al., 2017; Bahmani et al., 2018; Johnson et al., 2018a,b). Neural activity is either measured by spiking activity or local field potentials (LFPs), in order to encode sensory information (Csicsvari et al., 2003; Buzsáki and Draguhn, 2004; Liu and Newsome, 2006; Katzner et al., 2009; Whittingstall and Logothetis, 2009; Buzsáki et al., 2012). LFPs reflect the activity of a population of neurons based on spatially averaged synaptic activity (Buzsáki and Draguhn, 2004; Buzsáki et al., 2012; Jansen et al., 2014). They are continuous cyclic signals with various frequency bands. The low frequencies of the LFP as in delta, theta, alpha, and beta bands are a compound signal of slower events from a large population of cells. Therefore, the activity in the low-frequency bands of the LFP indicates the combination of neural activity across larger networks of neurons (Buzsáki and Draguhn, 2004). On the other hand, the high frequencies of the LFP such as low-gamma and high-gamma bands reflect higher local activity (Csicsvari et al., 2003; Liu and Newsome, 2006; Whittingstall and Logothetis, 2009).

The relation between spike times and the phase of LFPs has pointed out to several cognitive functions in various brain regions (Pesaran et al., 2002; Ray and Maunsell, 2010; Womelsdorf et al., 2010; Li et al., 2017), including the prefrontal cortex (Siegel et al., 2009), the visual cortex (Whittingstall and Logothetis, 2009), and hippocampus (Sirota et al., 2008; Cutsuridis and Hasselmo, 2011). For instance, the coupling between spikes to phases of LFP in the theta band encodes spatial memory in the hippocampus (Cutsuridis and Hasselmo, 2011). This spike–LFP phase relation, so-called spike-phase coupling (SPC), shows how activities of single neurons are harmonized for the averaged synaptic activity or LFPs to derive various cognitive functions. Measuring the coupling of single neuron’s spiking activities to the LFP is a method to investigate neuronal synchronization. Locking of spiking activities to LFP is a feature of such inter-neuronal synchrony. Several spike–LFP synchronization measures have been introduced in previous studies namely phase locking value (PLV) (Lachaux et al., 1999), spike field coherence (Grasse and Moxon, 2010), and pairwise phase consistency (Vinck et al., 2010, 2012). PLV, as one of the most important synchronization measures, represents the resultant length of the circular averaging of the instantaneous phases simultaneous to spikes. PLV vector is close to 1 for a trial when most spikes are coupled to a certain phase, and it is close to 0 when most spikes are spread across various phases (Lachaux et al., 1999). The shortcoming of PLV method is its bias on the number of spikes (Vinck et al., 2010, 2012; Zarei et al., 2018). To minimize this limitation, in general, an extra step is utilized in order to equalize the spikes of objective conditions for a certain count. Spike field coherence quantifies the synchronous activity between LFPs and spikes as a function of frequency. This method adds the power of LFP in different frequencies surrounding the corresponding spikes to the average LFP and normalizes this sum to the total number of spikes (Grasse and Moxon, 2010). Spike field coherence represents the coupling of spikes in regions around the LFPs. Hence, this method cannot exclusively represent the coupling of spikes to phase fluctuations. Lastly, the pairwise phase consistency method calculates the cosine of the absolute angular distance of the LFP phase across all possible pairs of spikes (Vinck et al., 2010). This method has a high variance for low spike counts and may yield negative values which are not physiologically justified. Therefore, this study employs SPC based on PLV method in order to uncover the effect of adaptation on the spike–LFP relation. Estimation of SPC through the PLV method reflects the phases of LFP for which spikes have occurred.

Neural adaptation is a common phenomenon which has been extensively observed in the mammalian sensory areas such as visual (Müller, 1999; Kayser et al., 2009), auditory (Ulanovsky et al., 2003; Dean et al., 2005; Anderson et al., 2009; Malmierca et al., 2009; Parto Dezfouli and Daliri, 2015), and somatosensory (Katz et al., 2006; Adibi et al., 2013) systems. Generally, adaptation tends to suppress neuronal activities in various sensory systems. Notably, suppression is only the case for a limited range of stimuli with repeated or prolonged stimulation (Dean et al., 2005; Adibi et al., 2013). Adapting to the environment as a result of the frequent representation of one stimulus, such as light, smell, or sound, is a vital brain function that the lack of it could be disturbing. Adaptation causes certain variations in neural properties in order to reduce attention to frequent stimuli. Particularly, it leads to an increase in the neural sensitivity against unexpected changes for deviance detection (Ulanovsky et al., 2003). Research on adaptation and change detection points to evoked potential signals in mismatch negativity studies, which includes human (Näätänen et al., 2007), primate (Javit et al., 1994), and cat (Csépe et al., 1987) experiments. The mismatch negativity is a component of event-related potential that occurs in response to a rare stimulus in a sequence. In the auditory system, two frequencies with almost similar responses are used in an oddball paradigm, represented by different probabilities. In some sequences, one tone is presented repeatedly as a standard stimulus and the other one is presented rarely and this role is swapped in other sequences. The difference between the responses in standard and rare conditions results in mismatch negativity which arises independent to the subject’s attention (Näätänen et al., 2007; Zhou et al., 2013). In recent years, the oddball paradigm has been extensively used in electrophysiological studies. As such, researchers have pinpointed interesting phenomena in the auditory cortical neurons, known as stimulus-specific adaptation (SSA) (Ulanovsky et al., 2003, 2004; Nelken and Ulanovsky, 2007; Parto Dezfouli and Daliri, 2015). Here, we used the term “adaptation” for “SSA” concept.

It is known that brain systematically suppresses neural responses to a repeated stimulus. This mechanism exists in various sensory areas of the brain which seems to suppress both field potentials and spiking activities. But how does this mechanism affect the relation between spike and LFP? We addressed this issue by investigating the SPC in the primary auditory cortex of the rat during an experiment consisting of two control and adapting conditions. In what follows, we will introduce our analytical and experimental methods in detail followed by the results of experiments and analyses. Finally, in the section “Discussion,” a thorough review of this work with specific references to key points will be presented.

## Materials and Methods

The procedure of surgery, experimental paradigm, data acquisition, and preprocessing of data are described in Parto Dezfouli and Daliri (2015). Here, we provide further details necessary to evaluate and present the current data analysis.

### Electrophysiological Recording

The data were recorded from the left auditory cortex (A1 area) of 15 adult male and female Wistar rats weighing 250–350 g. A linear multi-electrode array, consisting of four tungsten electrodes (FHC, 5M, United States; ∼5–10 μm tip diameter) were used for extracellular recording. The electrodes were directed into the cortex using a Microdrive (SM-21, Narishige, Japan). LFP and multi-unit activity (MUA) were recorded simultaneously using the USB-ME64-PGA recording system (Multichannel System, Germany). Furthermore, an online data visualization was utilized through a multichannel software known as “MCRack.” The recorded raw signals of each channel (with 10 kHz sampling rate) were initially pre-amplified by an eight-channel Miniature Preamplifier. Next, the amplified signals were band-pass filtered from 1 to 5 kHz and amplified with a gain of 1000 with a Programmable Gain Amplifier device. Finally, the recorded data were transmitted to the computer for subsequent off-line analyses.

### Experimental Paradigm

**FIGURE 1:**
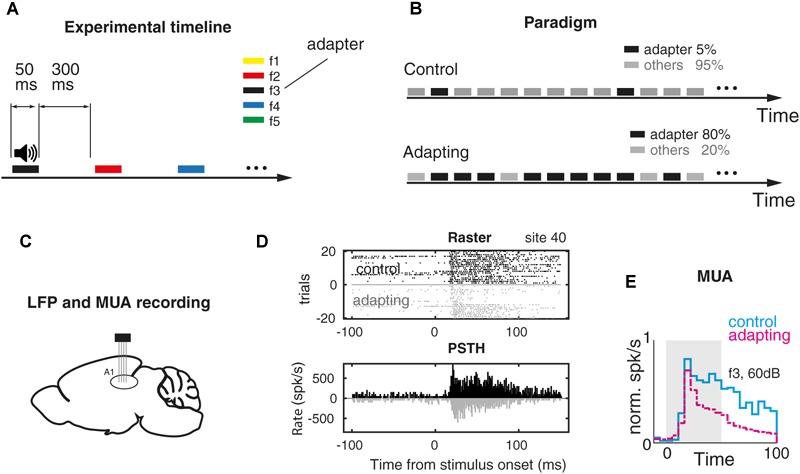
Adaptation experiment and MUA response. **(A)** Timeline of the auditory task. Pure tone stimuli were presented randomly for 50 ms and with 300 ms inter-stimulus interval. Stimuli were pure tones with a particular frequency and level out of five selected frequencies (f1–f5) and four intensities (40–70 dB SPL). **(B)** Two sequences of stimuli that were utilized for investigating the adaptation effect. In the first sequence (control) pure tones were presented with an equal probability. Each frequency–intensity combination was presented for 30 times. In the adapting sequence, the same combinations were presented with this a difference that the middle frequency at the level of 60 dB SPL (as the adapter) was presented with the probability of 80% of the whole sequence. **(C)** LFP and MUA were recorded from the primary auditory cortex of the anesthetized rat. **(D)** Raster plot and peristimulus time histogram (PSTH) of frequency f3 in 60 dB SPL intensity in a sample recording site. **(E)** Comparing PSTH of control vs. adapting conditions in the population of recording sites (*n* = 98).

Before the main task, in order to detect the selective neurons, broad-band noise bursts were presented with 300 ms duration and 500 ms inter-stimulus interval with an amplitude of 50 dB. For all four electrodes, only the channels for which average LFP amplitudes were large enough were recorded. For every recording, various arrangements of 11 frequencies (200 Hz to 20 kHz), with the frequency difference of *f* = 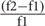 = 44% and seven intensities of 10–70 dB SPL at 10 dB steps, were considered in order to measure the frequency response area of every recording site. Each frequency–intensity combination was presented 10 times in a quasi-random sequence. Finally, based on the frequency response area of each single site, five selected frequencies around the characteristic frequency were considered for the main task. As a result, for each recording site, these five selected frequencies, namely f1–f5, in four higher intensities of 40–70 dB SPL in 10 dB steps, provided 20 frequency–intensity combinations. Figure 1A shows the timeline of the task in which these pure tone combinations were presented with 50 ms duration and 300 ms inter-stimulus interval. Because of several combinations of intensities and frequencies, the sequences had to be as short as possible in order to obtain comparable data from two paradigms in the same recording site. As such this study utilized a repetition of 2–3 Hz as an effective investigation of SSA paradigm (Taaseh et al., 2011; Shen et al., 2015; Takaura and Fujii, 2016). Figure 1B illustrates the main assessing task consisting of two control and adapting paradigms. For the control paradigm, 20 combinations consisting of five selected frequencies in four intensities were presented in a uniformly distributed manner. Each frequency–intensity combination was presented for 30 times. In the adapting paradigm, the same combinations were used somehow the middle frequency (f3) corresponding to the level of 60 dB SPL was considered as the adapter and the other combinations were assumed as its neighbors. Unlike the unbiased control paradigm, the adapting paradigm biased to the adapter. The adapter tone was presented four times per each presentation and therefore occupied 80% of all tone probability in this sequence. For instance, in the control paradigm with 600 trials (20 combinations in 30 times), the stimuli were presented with the same probability of 5%. While in the adapting paradigm with 2850 stimuli, the adapter was presented 2280 times (80%) and all other 19 combinations were presented for 570 times (20%). As a result, the adapter stimulus (as the target condition of this study) was presented with the probability of 5 and 80% in control and adapting sequences, respectively. In order to ensure the stability of recording conditions, the first paradigm was presented once before and once after the adapting paradigm. The sites which responded with >30% variation were excluded.

### Data Analysis

The LFP and MUA were recorded from the primary auditory cortex of anesthetized rats using a simultaneous four-channel recording. For this study, we considered only the sites with responsive activity for both MUA and LFP. Overall 98 sites were selected for the main analyses. Each 50 ms stimulus presentation with its following 300 ms delay interval was considered a trial. Accordingly, every recording site consisted of ∼30 and 2280 trials of the characteristic frequency (f3) in the control and adapting paradigms, respectively, where the number of 2280 trials is based on four iterations for every 30 trials of 19 combinations. All analyses were carried out in MATLAB 2016b (Mathworks, Natick, MA, United States). For LFP, the raw data were passed from a low-pass filter with a cutoff frequency of 300 Hz. Likewise for MUA analyses, first the raw signals were filtered using a band pass filter with cut-off frequencies of 300 and 3000 Hz. Subsequently, spike times were detected by a threshold crossing method. Ultimately, all responses were aligned to the stimulus onset and their baselines were corrected (Dezfouli et al., 2014). In order to correct for the baseline, as close as possible to the onset of the response, the average response of the first 5 ms duration from the stimulus onset was subtracted from it. The shortest response latencies were consistently observed to be longer than 8 ms. The standard error of the mean (SEM) was considered as a criterion for measuring the variability of the response.

The time window of 0–100 ms with respect to stimulus onset was selected as the duration of the analysis. To remove the 50 Hz noise and for the purpose of filtering the LFPs in different frequency bands, a band-pass and non-causal finite impulse response (FIR) filter was used. The traditional bands were considered for the analysis of LFP signals, namely delta (δ; 1–4 Hz), theta (𝜃; 5–8 Hz), alpha (α; 9–12 Hz), beta (β; 13–30 Hz), low-gamma (γ_L_; 31–70 Hz), and high-gamma (γ_H_; 70–120 Hz). Since we found the coupling of the spike to LFP phase in beta range, we focused our analyses within the beta band (13–30 Hz), but the frequencies up to 120 Hz were also analyzed. Likewise, we filtered the beta range of LFPs into separate 4 Hz windows. For each frequency-based window, SPC was computed for the control and the adaptation conditions. Notably, the sites with a responsive spiking activity to stimuli and LFPs with a negative wave in deep layers were selected for SPC analysis. Moreover, in order to avoid edge effects, created by the cut-off at the starting and end of an LFP time section, we added 900 ms data from the same trial to both ends of the LFP (±450) and later eliminated the corresponding parts from the filtered signal.

### Spike-Phase Coupling (SPC) Based on the PLV Method

As noted before, SPC shows how activities of neurons are coordinated for the averaged synaptic activity or LFPs. Here, we employed PLV to measure SPC. PLV method measures the strength of consistency or locking of phases in spike times, by calculating the angular summation between phases of LFP in spike times. The amplitude of PLV shows the SPC strength and its angle reflects the phases of LFPs for which spikes have occurred. To evaluate PLV in selected frequency bands, the Hilbert transform was utilized for the analytic signal of LFP. The Hilbert transform is defined as,

$$\mathrm{HT}(x(t))=\frac{1}{\;\pi }P\int_{-\infty }^{\infty }\frac{x(\tau )}{t-\;\tau }\;d\tau$$

It converts a real-valued signal (x(t)) into a complex analytical signal (HT(x(t))) where *P* is the Cauchy principal value of the singular integral. Next, instantaneous phases ϕ(t) were obtained by calculating the angles corresponding to the aforementioned analytic signal,

$$\phi (t)=\arctan\left(\frac{\mathrm{HT}(x(t))}{x(t)}\right)$$

Furthermore, PLV method is described by the following formula:

$$\mathrm{PLV}=\;\frac{1}{N}\left|\sum_{n=1}^{N}\exp\left(j\phi \right)\right|$$

where *N* is the number of spikes and φ is the LFP phase at the times of spike occurrence.

It is worth to note that one limitation of SPC estimation is that the value of SPC is dependent on spike numbers. For instance, if one compares two conditions with different spike numbers, it is more likely that the condition with a larger spike number offers less SPC. This issue was addressed in two recent studies on SPC (Vinck et al., 2012; Zarei et al., 2018). To overcome this issue we equalized the number of spikes in the control and adapting conditions. Hence, after identifying a threshold for the average spike number, trials with spike numbers < threshold were removed and spikes in trials with spike numbers > threshold were reduced (removed randomly) to the threshold value. Notably, the LFP amplitudes for each recording site were normalized by subtracting the mean and dividing the result by the standard deviation in order to create normalized LFP signals. Normalization was performed to set aside the possibility of LFP power effect on the SPC strength.

### Spike-Triggered Average (STA) LFP

The spike-triggered average (STA) LFP is a quantity which links a spike train with the LFP, recorded simultaneously. It indicates the average LFP value chosen at the times of the occurrence of spikes. STA is calculated by averaging the LFP amplitude in a trace surrounding spiking times. In this study, the LFP trace from -20 to 20 ms relative to the spike times was averaged to estimate the STA where this sum was subsequently divided by the total number of spikes. Notably, the STA would produce a flat result for independent neuronal activities. Otherwise, the result could indicate the coupling of spikes to a particular phase of LFP.

## Results

To elucidate the effect of adaptation on SPC, we performed an auditory experiment consisting of two control and adaptation conditions (Parto Dezfouli and Daliri, 2015). The timeline of the experiment and the two control and adapting paradigms are shown in Figure 1A,B. Twenty combinations of pure tones were used in the experiment. In the control paradigm, they were presented randomly with a similar probability that each combination of pure tones occupies 5% of the sequence. In the adapting paradigm, the same stimuli were presented randomly with this difference that in the adapting paradigm the middle frequency (f3) at the level of 60 dB SPL (as the adapter) occupied 80% of the total sequence. The rest of the combinations occupied 20% of this sequence. We collected LFP and MUA from 98 recording sites over the primary auditory cortex of 15 anesthetized rats while they were presented by auditory pure tones in two conditions (Figure 1C – see the section “Materials and Methods**”**). The original signal of an example recording site (site 14; control paradigm) before its conversion to LFP and spiking activity is shown in Supplementary Figure S1. The raster plot and peristimulus time histogram (for 20 trials) of the characteristic frequency related to a single recording site (site 40) were shown for the control and adapting conditions, separately (Figure 1D). Comparison of the population spiking activity between control and adapting conditions, in line with previous studies (Ulanovsky et al., 2003; Nelken and Ulanovsky, 2007), shows a suppression in the spiking activity due to adaptation (Figure 1E).

### Spike-Phase Coupling Suppressed by Adaptation

**FIGURE 2:**
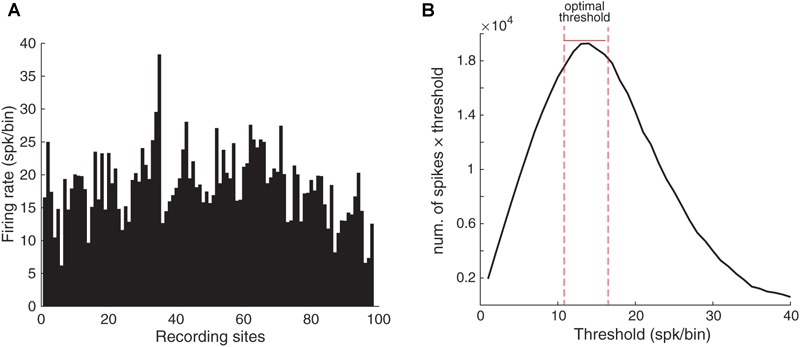
Selecting the optimal threshold. **(A)** The average number of spikes in each electrode during the analysis period (100 ms). The *X*-axis shows the site indices and the *Y*-axis shows the spike rate. **(B)** The optimal spike count threshold for computing SPC. The *X*-axis indicates different values for the spike count threshold and *Y*-axis indicates the total spike count considered for each threshold (*T*). The optimal threshold (*T* = 14 spk/bin) produced the maximum spike count from the neural data. The bin is the 100 ms window from stimulus onset.

Comparison of the SPC of a particular stimulus between control and adapting conditions indicated that SPC was reduced due to adaptation (SPC of adaptation < control). We employed PLV to calculate SPC and since it is believed that the PLV method is biased to the spike count, first one needs to equalize the spike counts of trials within each recording site. To this end, we determined the average number of spikes for each recording site (Figure 2A). Using this average, it is possible to estimate the value of spike rates for each recording site to drop extra spikes. Indeed, this is the easiest way to calculate the spike count threshold (*T*) and is frequently preferred by researchers while it is not optimal.

Here, we used an optimal thresholding method which results in an optimal compromise between the number of spikes and trials. We assumed different spike count thresholds for finding the optimal threshold. Due to PLV’s bias on the spike count, we first balanced the spike count across different sites. To obtain the optimal threshold for the spikes number, we computed the total spike count from the neural data that would be remained after utilizing different thresholds (Figure 2B). Accordingly, the number of *T* = 14 spk/bin was selected as the optimal threshold value. Here, the bin is the 100 ms window from stimulus onset. Consequently, the trials with the number of spikes below the threshold were removed. Also, the trials with spikes number higher than the threshold were equalized relative to spike number to the threshold value by removing the additional spikes randomly. Thus, all resulting trials had the same spike rates and we could use an existing method such as PLV in order to calculate the SPC.

**FIGURE 3:**
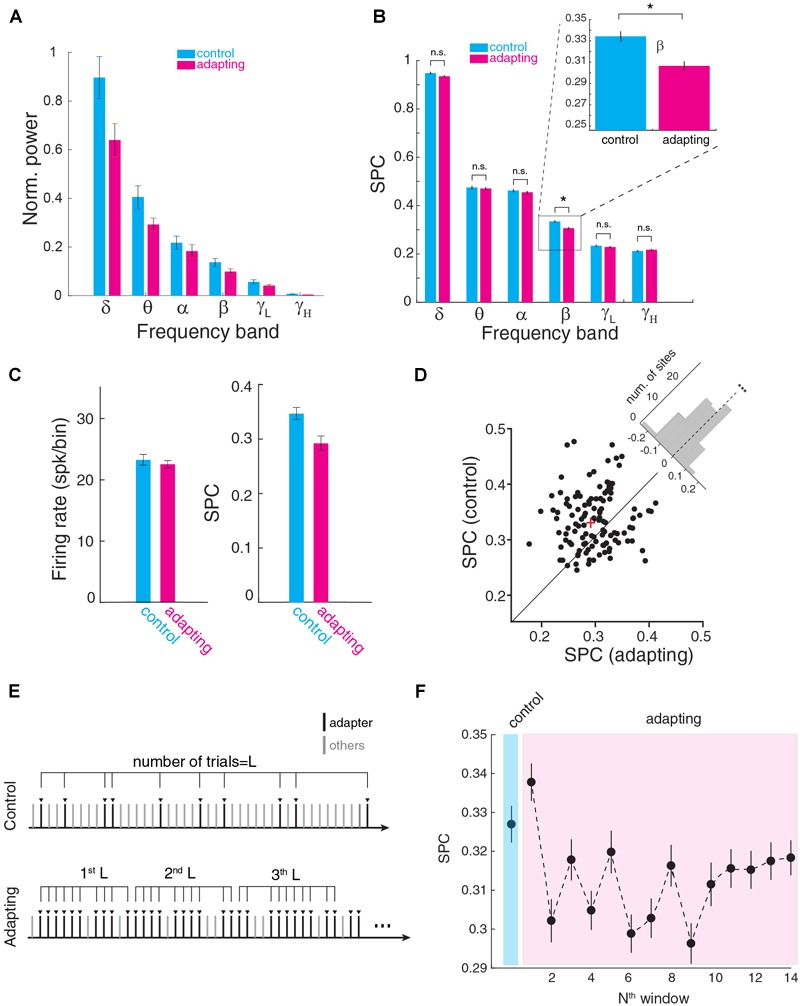
Spike-phase coupling at different frequency bands. **(A)** Power induced in six frequency bands for control and adapting conditions. The average band power was normalized to the maximum power. Significant power reduction observed in all frequency bands except alpha band. Maximum power difference between control and adapting condition arose in delta band and the minimum was in alpha band. **(B)** SPC strength for control and adapting conditions averaged across neurons for different frequency ranges of delta (δ; 1–4 Hz), theta (𝜃; 5–8 Hz), alpha (α; 9–12 Hz), beta (β; 13–30 Hz), low-gamma (γ_L_; 31–70 Hz), and high-gamma (γ_H_; 70–120 Hz). SPC strength is shown to be significantly different for the two conditions within the beta band (*p* < 0.0001; Wilcoxon rank sum test). **(C)** Comparison of SPC between the f3 condition of adapting sequence and a condition with similar spike rate in the control condition. The SPC in the adapting condition reduces (right panel) while the spiking activity was similar (left panel). **(D)** Scatter plot of the SPC strength (control vs. adapting) within the beta band (13–30 Hz) for all recording sites. The “cross” sign marks the average SPC (control vs. adapting) of the population of the recording site. The histogram in the upper right shows the distribution of recording sites toward two objective conditions, namely control and adapting (*p* < 0.001; *t*-test, *n* = 98, mean = –0.029, std = 0.071). It illustrates the distribution of SPC difference (SPC_adapting_-SPC_control_) across all recording sites. Each data point corresponds to a recording site. **(E)** Schematic of division of the adapting trials into partitions with the same length (L) based on the number of test trials in the control sequence (L). **(F)** Time course of adaptation effect on SPC. For all adapter trials in the adapting sequence, the data were partitioned based on its trials’ number in the control sequence (L trials in each bin). SPC strength in the adapting sequence is shown to be lower than the control sequence as the desired window increases (exponentially descends).

We next measured the SPC strength within six frequency bands; delta to gamma. First, we calculated the power-induced LFP signal in these frequency bands and compared the band power between control and adapting conditions (Figure 3A). Results show a significant reduction of LFP power almost in all frequency bands except alpha band (*p* < 0.05, *t*-test). On the other hand, as shown in Figure 1E and also previous reports (Taaseh et al., 2011; Parto Dezfouli and Daliri, 2015) adaptation reduces the firing rate. Higher rate of activity leads to less SPC strength based on PLV (Supplementary Figure S2). Accordingly, to minimize any effect of LFP power or firing rate difference on SPC values, along with equalizing spike rate, we normalized LFPs and considered just their phase feature. Next, we estimated the SPC strength in each frequency band (Figure 3B). Figure 3B shows the SPC strength for the control and adapting conditions which were averaged across recording sites. Here, SPC strength was significantly different for the control and adapting conditions within the beta (13–30 Hz) band (p << 0.01; Wilcoxon rank sum test; *p* < 0.05; Bonferroni correction). Other bands showed no significant SPC difference between the control and adapting conditions (δ; *p* = 0.14), theta (𝜃; *p* = 0.61), alpha (α; *p* = 0.52), low-gamma (γ_L_; *p* = 0.57), and high-gamma (γ_H_; *p* = 0.35).

To investigate whether the phase decoupling is a consequence of reduced responsiveness of neurons (both in terms of LFP and spiking activity), or is governed by additional mechanisms, we compared the SPC of adapter stimulus under adaption condition with the SPC for a stimulus under a control condition with the matched firing rates. By comparing the lower intensities (intensity at the level of 50 dB) in the control sequence which have similar firing rate as that of the adapting condition, the coupling was less for adapting condition while their firing rates were matched (Figure 3C). This result shows that the adaptation-induced decoupling is mediated by a separate mechanism than suppression of excitability and responsiveness of individual cells. Subsequently, the SPC strength of control and adapting condition was compared across all recording sites. Figure 3D shows the SPC of all recording sites (*n* = 98) within the beta band (13–30 Hz). Each dot represents the SPC strength of the control vs. adapting condition for a recording site. The histogram in the upper right shows the distribution of two conditions. The results show that across the sites, SPC strength for the control condition is significantly higher than adapting condition (*p* < 0.001; *t*-test, *n* = 98, mean = -0.029, std = 0.071).

We further performed a systematic examination of the time-course of the adaptation effect on the SPC strength. For this purpose, as depicted in Figure 3E, we partitioned the probing trials in the adapting paradigm based on its number in the control sequence (*L* = 30), namely [1: L], [L: 2L], etc. Next, SPC was computed for L control probe trials and compared with 14 L portions of adapting trials (Figure 3F). The result shows that a reduction of SPC strength emerges from the second part of L trials in the adapting paradigm. This value converges to a stable value from ∼10th partition after some fluctuations.

### Spikes Are Coupled to the Phase of LFPs Within the Beta Band

Previous studies have revealed that the spike rate is attenuated in the adapted condition compared to the control condition (Taaseh et al., 2011; Parto Dezfouli and Daliri, 2015). This study suggests that adaption causes SPC to reduce. This reduction occurs in the coupling of spikes to LFP phase within the beta band.

**FIGURE 4:**
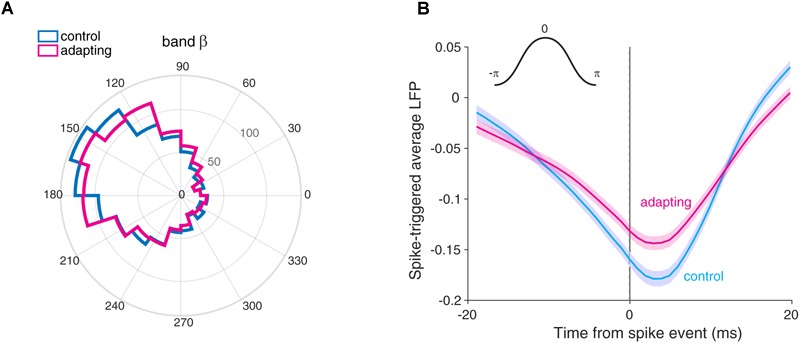
The preferred phase of LFP. **(A)** The histogram of phases for the preferred LFP phase in control (cyan) and adapting (magenta) conditions within the beta band (13–30 Hz). The mean locking phases are 0.91 and 0.88 rad (165° and 158°) for control and adapting conditions, respectively. **(B)** Spike-triggered average (STA) of normalized LFP across sites for control (cyan) and adapting (magenta) conditions. STA curves show the spikes are coupled to the ∼160° of the phase of LFP for two conditions. The inset in the upper section illustrates the phase values in a cycle. Shade areas depict standard error of mean across all recording sites.

To examine the relation of this reduction of spikes to LFP phase, we first tested if the preferred phase for two conditions is matched. We defined the preferred phase as the phase in which spikes prominently occur (α) and consequently give the most SPC value. Accordingly, anti-preferred phase was defined as the phase with 180° distance from the preferred phase (180 - α). For this purpose, the histogram of LFP phases in spike times was computed for both conditions. Figure 4A shows the histogram of LFP phases in the beta band across sites for both control and adapting conditions. As depicted, the distribution of phases across recording sites differs significantly from the uniform distribution in both conditions. The mean locking phase is 2.89 and 2.76 rad (165°, 158°) for control and adapting conditions, respectively, and there is no statistically significant difference between them (*p* = 0.3, *t*-test). This suggests that spiking activity in the auditory cortex tends to occur more frequently at a similar phase within the beta band (13–30 Hz) of LFPs, independent of adapting or control conditions. The results of the SPC for each 4 Hz frequency window from 1 to 60 Hz are shown in Supplementary Figure S3A. Considering 4 Hz frequency windows shows this phase consistency occurs prominently in 16–20 Hz of the beta band (Supplementary Figure S3B).

We also measured the preferred phase of LFP based on STA method. To this end, we averaged the LFPs within a window (±20) around each spike occurrence. As depicted in Figure 4B, we computed the STA across all recording sites (*n* = 98) for control (cyan) and adapting (magenta) conditions. The difference between the peak and trough for the control (cyan) and adapting (magenta) conditions shows that the SPC for the control condition is larger. Moreover, Figure 4B shows spikes coupled to the falling phase (∼160°) (consistent with Figure 4A) of LFP for both conditions.

### Spikes Are Suppressed From the Preferred Phase of LFP

**FIGURE 5:**
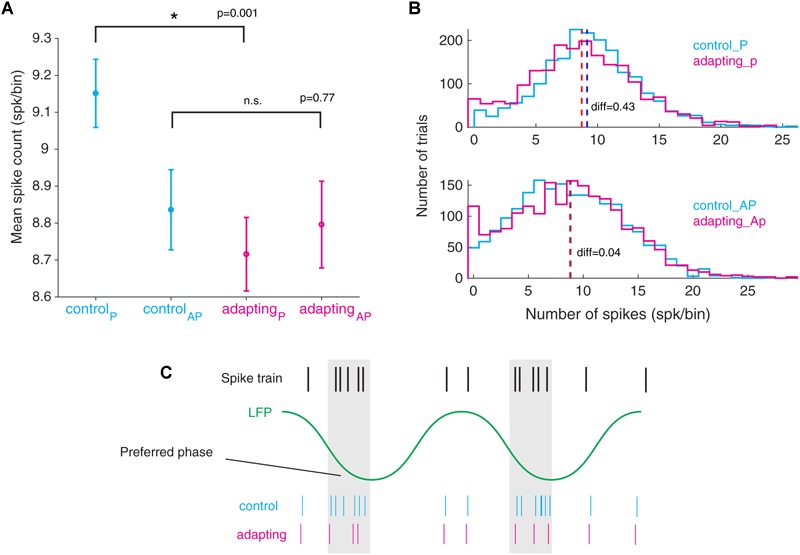
Declining in the number of spikes from the preferred phase of LFP during adaptation. **(A)** Mean standard error of spike counts at both the preferred and the anti-preferred LFP phase for the control vs. adapting conditions. All LFP phases are divided into two groups of preferred phase ± π and anti - preferred phase ± π where the spike counts in these two groups are compared. **(B)** Histogram representation of spike counts in the preferred phase and anti-preferred phase for control (cyan) and adapting (magenta) conditions, respectively. **(C)** Schematic illustration of spike suppression from the preferred phase of LFP in the adapting condition compared to the control condition.

Subsequently, we investigated the relation between the spike suppression and the LFP phase. To this end, we compared the spike counts of the preferred phase for control and adapting conditions. Likewise, we conducted this procedure for the anti-preferred phase (Figure 5A). As such, one could realize whether the suppression of spikes occurs in the preferred or anti-preferred phase. The difference between the spike counts before and after adaptation (control and adapting conditions) for each of the preferred phase and anti-preferred phase depicts the number of suppressed spikes for the corresponding phase. Results show that a significant reduction of spikes occurs in the preferred phase (Wilcoxon rank sum test, *p* < 0.001) while no visible difference is observed for the anti-preferred phase. Figure 5B shows the distributions of preferred and anti-preferred phases for control (cyan) and adapting (magenta) conditions, respectively. Comparing the values of mean spike count in the population of sites (difference=∑ spk_control_ -∑ spk_adapting_) shows that the suppression of spikes occurs dominantly in the preferred phase of LFP. Figure 5C depicts how the preferred phase in LFP is defined according to the spike train and depicts the relation of spiking activity in control and adapting conditions.

## Discussion

This study reported that SSA suppresses the spike to LFP phase coupling most prominently in beta range. It also revealed that the adaptation-induced spike reduction occurred for the preferred phase of LFP.

As noted before, SPC shows how activities of spikes are coordinated in the LFPs for different cognitive functions such as sensory coding, attention, working memory, and adaptation (Baker et al., 1999; Kayser et al., 2009; Cutsuridis and Hasselmo, 2011; Muthukumaraswamy, 2011; Mendoza-Halliday et al., 2014; Ruff and Cohen, 2014; Fazlali et al., 2016; Ding et al., 2017; Bahmani et al., 2018). This coupling has also been found to be reduced with attention in visual area V4 (Fries et al., 2001). Also, it is indicated that the correlation in spiking activity between neurons is reduced by attention (Cohen and Maunsell, 2009). Likewise, the coupling has been documented to encode working memory contents in spike–LFP relation within an area (Bahmani et al., 2018) as well as phase–phase synchrony between two areas (Johnson et al., 2018a,b). It is worth to note that the high frequencies of the LFP such as low- and high-gamma bands reflect more local activity (Csicsvari et al., 2003; Liu and Newsome, 2006; Whittingstall and Logothetis, 2009). On the other hand, the low frequencies of the LFP such as delta, theta, alpha, and beta ranges are a compound signal of slower events from a very large population of cells. Therefore, the activity in the low-frequency bands of the LFP indicates the combination of neural activity across larger networks of neurons (Buzsáki and Draguhn, 2004). As a result, synchronization of spiking activity with LFP in low frequency ranges appears better and with more strength, in different cortical areas, and through various cognitive functions.

The length of the PLV vector represents an estimation of the strength of SPC. It computes a value between 0 and 1 for a given number of spikes for a trial. The limitation of the PLV method is its bias on the number of spikes (Zarei et al., 2018) and studies that use this method, equalize the spikes at a certain count. Therefore, to compute the SPC by this method an equalizing plan was used in order to obtain the spike counts based on a threshold. This study utilized an optimal thresholding scheme which provided an optimal compromise between the number of spikes and the number of trials (Zarei et al., 2018). As such, the trials whose number of spikes were below the threshold were removed, and the trials with their spikes higher than the threshold were equalized to it. It may be argued that how does this method affect the number of trials per condition (control vs. adapting) individually? To address this issue, as depicted in Figure 2 there is a tradeoff between the threshold value and the number of remaining trials after spike-count equalization. The optimal thresholding selected a threshold somehow the minimum number of trials needs to be removed. Supplementary Figure S4A shows the relation between trial numbers in each condition for 10 threshold values (between 1 and 46). It shows that with the threshold value of 14 (spk/bin) no significant difference is visible between two control and adapting conditions. Moreover, we equalized the number of trials between control and adapting conditions before SPC calculation. The SPC strength in beta band shows a significant difference from chance level SPC in threshold values between 7 and 30 spk/bin (Figure 3 and Supplementary Figure S4B). Hence, calculation of SPC by the PLV method revealed the phases of the LFP that spikes have occurred.

The main purpose of this study was to investigate the role of synchrony in neural adaptation mechanism. Here, we examined the effect of adaptation on SPC over the primary auditory cortex area. Commonly, adaptation suppresses the neuronal activities, namely spikes and LFPs in sensory systems. To assess the effect of adaptation on the relation of spikes to LFP phases (SPC), this study calculated the strength of locking between spikes and field potentials by quantifying PLV (Lachaux et al., 1999) in control and adapting conditions.

Recently, the effect of cognitive functions such as adaptation and attention on population synchrony has reported in various brain areas. Adibi et al. (2013) showed that stimulus presentation reduced individual neuron trial-to-trial response variability (captured by Fano factor) and correlations in the population response variability (noise correlation). It has been shown that adaptation conveyed the neuronal operating scheme to lower rates with higher Fano factor and noise correlations (Adibi et al., 2013). Furthermore, Gutnisky and Dragoi (2008) showed that noise correlations are independent of stimulus orientation and caused a strong reduction in correlations after adaptation. Mitchell et al. (2007) found that correlations in spiking averaged are significantly reduced with attention directed into the receptive field. Consistent with previous research in the response function of cortical neurons, our results suggest that the SPC within the beta band is significantly reduced with adaptation (Adibi et al., 2013, 2014; Ponce-Alvarez et al., 2013).

Our results indicate that spikes are coupled to the falling phases of LFP in the primary auditory cortex of the rat (Figure 4). This finding is consistent with the previous studies in a different cortical area of the macaque (Lakatos et al., 2008; Whittingstall and Logothetis, 2009). This SPC within the beta band frequency is suppressed by neural adaptation. Furthermore, we found that the reduction of spikes in adaptation occurred at the preferred phase of LFP rather than the anti-preferred phase, or other phases (Figure 5). This result provides valuable information toward a better understanding of the underlying neural mechanism of adaptation and could be utilized in the context of biological neural modeling. In addition, this finding may suggest the information coded by single neurons fluctuates relative to the preferred phase of LFP. As a result, the preferred phase of LFP may play a key role in coordinating neuronal spiking activity in different brain functions.

## Data Availability

The datasets generated for this study are available on request to the corresponding author.

## Ethics Statement

Research with animal subjects represents a small but indispensable component of neuroscience research. The scientists in this study are aware and are committed to the great responsibility they have in ensuring the best possible science with the least possible harm to the animals (Calapai et al., 2017). The main goal of the current study was to elucidate the effect of adaptation on SPC as a crucial component of measuring brain function. All experiments were carried out in Iran Neural Technology Center (INTC) at Iran University of Science and Technology (IUST) with the approval of, and using methods conforming to the standards of the ministry of health and medical education. The animals are maintained in the cages in facilities of the Neural Technology Center. The animal care and use committee of Neuroscience Research Laboratory, IUST approved all surgical procedures and experimental protocols in strict accordance with the recommendations in the Guide for the Care and Use of Laboratory Animals of the National Institutes of Health. All surgery was performed under urethane pentobarbital anesthesia, and all efforts were made to minimize suffering. At the end of the experimental period, the animal was sacrificed under ether anesthesia (Parto Dezfouli and Daliri, 2015).

## Author Contributions

MPD, MZ, MJ, and MRD contributed to the concept of the work. MPD performed the experimental recording. MPD and MZ analyzed the data. All authors contributed to drafting, revising, and finalizing the manuscript. MRD and MJ supervised the work.

## Conflict of Interest Statement

The authors declare that the research was conducted in the absence of any commercial or financial relationships that could be construed as a potential conflict of interest.

## Supplementary Material

Figure S1 An example of original recording and spikes to phase relation. **(A)** Data were recorded with 10 kHz sampling rate to cover both LFP and spike activities. The lower panel shows the raw data of a sample recording site (site 14-control paradigm) vs. time. The upper panel indicates ∼700 ms recording time with the presentation of two pure tones. **(B)** Raw LFP, filtered LFP in beta band, its extracted phase in this band for a sample trial, and spike event times for a three sample trials with the same LFP oscillations in beta band.

Figure S2 The SPC reduction due to adaptation is independent of firing rate. By dividing the trials of adapting sequence into two groups; lower than median and higher than median firing rate, we evaluated the effect of firing rate on SPC value. Results show that the low firing rate (LFR) group leads to more SPC value than high firing rate (HFR) group. Two conditions were selected from the adapting sequence.

Figure S3 SPC for 4 Hz segments and per 4 Hz stepping size. **(A)** Based on Bonferroni criterion (0.05/15–0.003), the SPC within 16–20 and 20–24 Hz shows significant difference between control and adapting conditions. **(B)** Polarity maps of three sample bands (4–8, 16–20, and 48–52 Hz) that indicate dispersed phase for other bands except beta band.

Figure S4 Effects of the threshold value. **(A)** The relation between remained trial numbers in each control and adapting conditions for 10 threshold values (from 1 to 46) in the optimal thresholding plan. It shows that almost the same number of trials were decreased from both control and adapting conditions. Notably, we also equalized the number of trials between control and adapting conditions for SPC calculation. **(B)** SPC strength for control and adapting conditions compared to the chance level of SPC relative to changing of the threshold value.
