# Supplementary figures and images for: Stimulus-Specific Adaptation Decreases the Coupling of Spikes to LFP Phase
Source: Front Neural Circuits. 2019 Jul 3;13:44. doi: 10.3389/fncir.2019.00044 (PMC6616079; doi:10.3389/fncir.2019.00044)

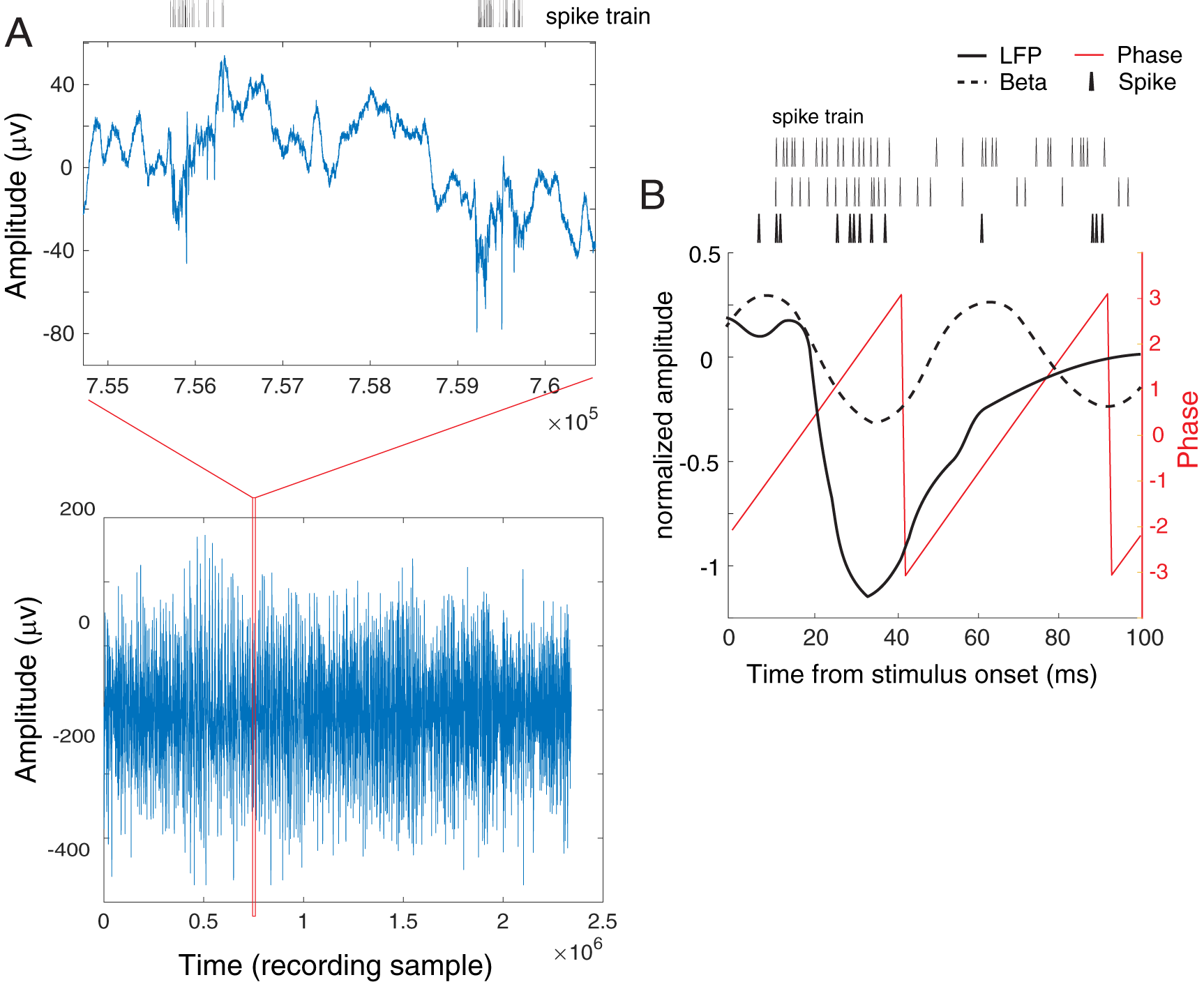

Supplement: Figure S1 — An example of original recording and spikes to phase relation. (A) Data were recorded with 10 kHz sampling rate to cover both LFP and spike activities. The lower panel shows the raw data of a sample recording site (site 14-control paradigm) vs. time. The upper panel indicates ∼700 ms recording time with the presentation of two pure tones. (B) Raw LFP, filtered LFP in beta band, its extracted phase in this band for a sample trial, and spike event times for a three sample trials with the same LFP oscillations in beta band. [file Image_1.TIF]

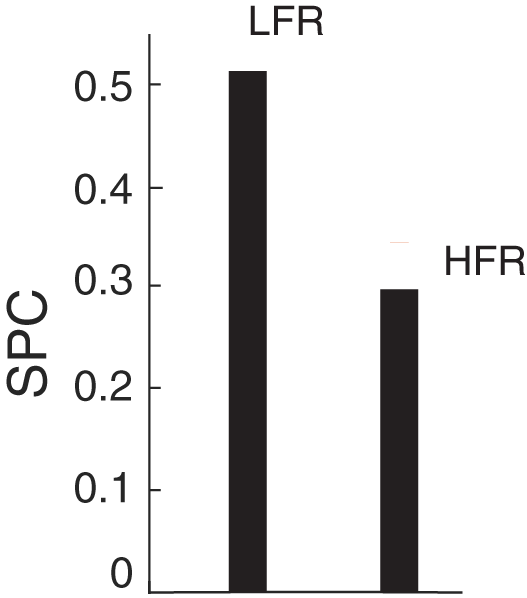

Supplement: Figure S2 — The SPC reduction due to adaptation is independent of firing rate. By dividing the trials of adapting sequence into two groups; lower than median and higher than median firing rate, we evaluated the effect of firing rate on SPC value. Results show that the low firing rate (LFR) group leads to more SPC value than high firing rate (HFR) group. Two conditions were selected from the adapting sequence. [file Image_2.TIF]

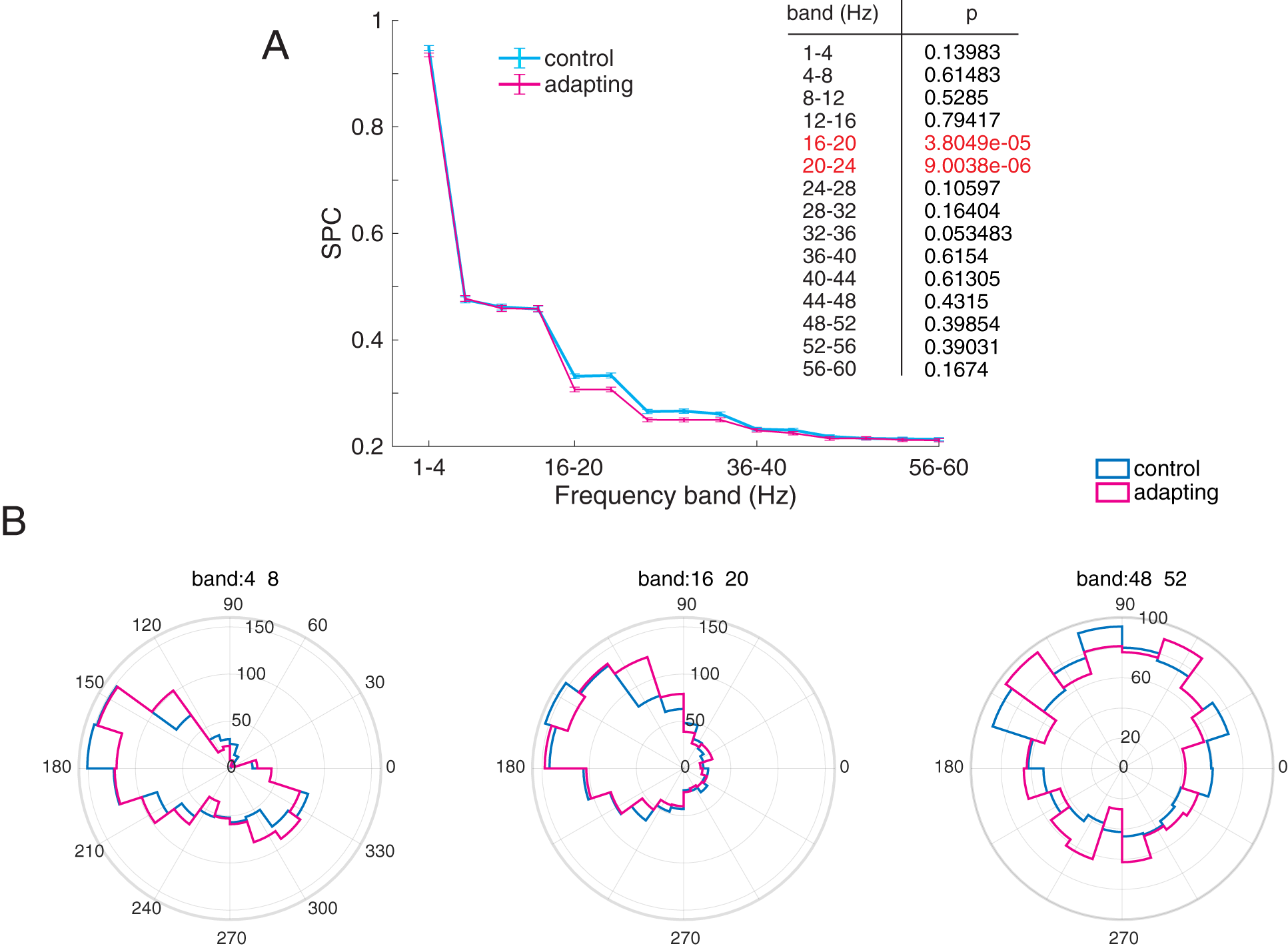

Supplement: Figure S3 — SPC for 4 Hz segments and per 4 Hz stepping size. (A) Based on Bonferroni criterion (0.05/15–0.003), the SPC within 16–20 and 20–24 Hz shows significant difference between control and adapting conditions. (B) Polarity maps of three sample bands (4–8, 16–20, and 48–52 Hz) that indicate dispersed phase for other bands except beta band. [file Image_3.TIF]

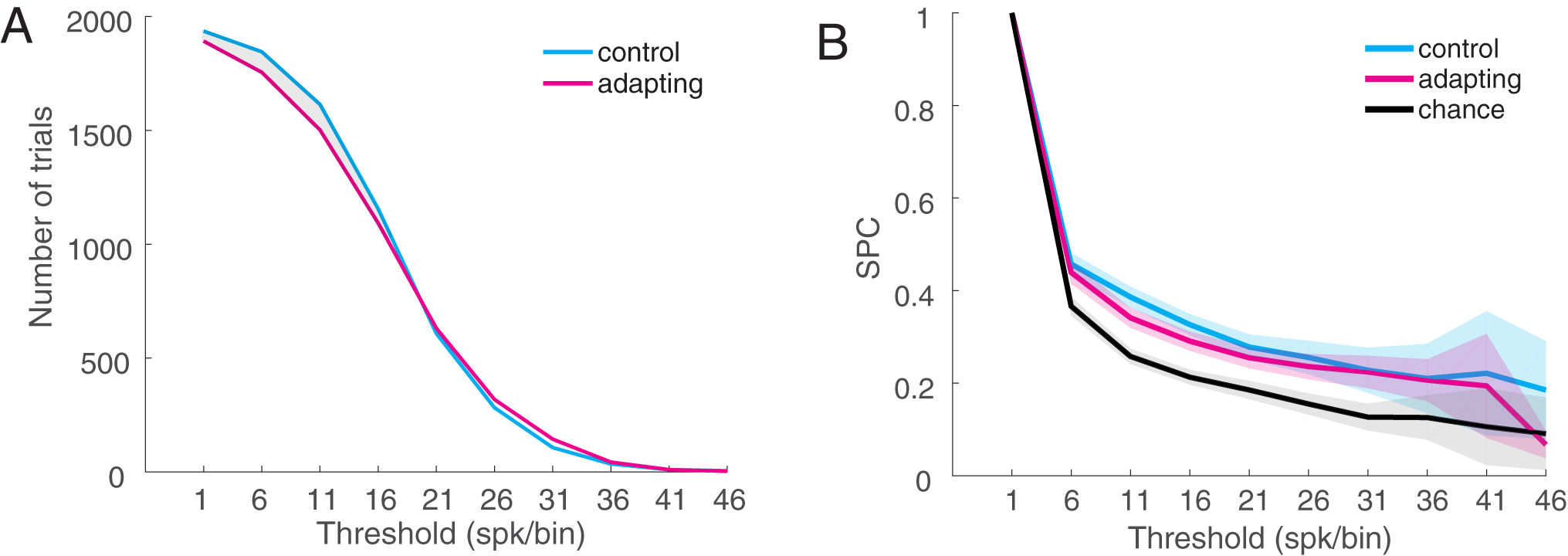

Supplement: Figure S4 — Effects of the threshold value. (A) The relation between remained trial numbers in each control and adapting conditions for 10 threshold values (from 1 to 46) in the optimal thresholding plan. It shows that almost the same number of trials were decreased from both control and adapting conditions. Notably, we also equalized the number of trials between control and adapting conditions for SPC calculation. (B) SPC strength for control and adapting conditions compared to the chance level of SPC relative to changing of the threshold value. [file Image_4.TIF]
